# Enhancing Dressing Clinic Nurses' Knowledge in Breast Reconstruction Care: An Educational Intervention Study

**DOI:** 10.7759/cureus.105714

**Published:** 2026-03-23

**Authors:** Niall O'Hara, Kuljyot Bajaj, Doraisami Mohan

**Affiliations:** 1 Plastic and Reconstructive Surgery, Russells Hall Hospital, Birmingham, GBR

**Keywords:** breast reconstruction, diep, educational intervention, microsurgery, service development

## Abstract

The deep inferior epigastric perforator (DIEP) free flap is the gold standard for autologous breast reconstruction. We identified a knowledge gap among dressing clinic nurses in a unit establishing a DIEP reconstruction service. A quality improvement project was undertaken to deliver a structured educational programme for nursing staff involved in postoperative follow-up of these patients. The intervention comprised formal teaching, dissemination of standardised care pathways, case-based discussions, and opportunities for theatre observation. Thirty-seven participants completed pre- and post-intervention questionnaires assessing self-reported confidence and knowledge across five domains, with additional qualitative feedback. Baseline confidence varied, particularly regarding flap assessment, recognition of complications, and patient counselling. Following the intervention, there was a statistically significant improvement in confidence across all five domains (p<0.001). Nearly all participants (97%) recommended the teaching programme. Qualitative feedback highlighted an improved understanding of flap anatomy, enhanced confidence in escalation of concerns, and greater engagement within the multidisciplinary team. Theatre observation was reported as particularly impactful. Our structured educational intervention significantly improved dressing clinic nurses' confidence and perceived competence in managing patients following DIEP reconstruction. This approach offers a practical framework to support safe service development and multidisciplinary integration when introducing new microsurgical services.

## Editorial

The deep inferior epigastric perforator (DIEP) free flap is widely accepted as the gold standard for autologous breast reconstruction, providing improved patient outcomes while minimising donor-site morbidity compared with transverse rectus myocutaneous (TRAM) flaps [1]. Successfully establishing any new microsurgical service relies on effective multidisciplinary collaboration, particularly with nursing teams responsible for postoperative care.

We introduced a new DIEP reconstruction service in a hospital with no previous experience of free tissue transfer surgeries, revealing a gap in dressing clinic nurses' familiarity with postoperative flap management. Their role in wound assessment, recognition of complications, and patient education made this knowledge gap clinically significant. Our quality improvement project aimed to deliver a structured educational intervention and assess its impact.

Our project was conducted in accordance with local governance guidelines. Informed consent was gained from all participants. All plastic surgery and dressing clinic nurses involved in the follow-up of breast reconstruction patients were invited to participate voluntarily.

The educational intervention consisted of structured teaching sessions on DIEP flap anatomy and standardised postoperative care pathways; case-based discussions with clinical images of normal and compromised flaps; and roleplay scenarios for patients' frequently asked questions. We also created opportunities for outpatient staff to attend the operating theatre to observe DIEP surgeries.

Teaching was delivered during prescribed nursing teaching hours on hospital audit days by members of the plastic surgical team. Participants completed anonymous pre- and post-intervention questionnaires assessing self-perceived confidence and knowledge across five domains: flap observations, donor-site care, patient communication, recognition of complications, and escalation of concerns. Each domain was rated on a Likert scale. Qualitative feedback was also collected through invited free-text feedback.

All participating nurses (n=37) completed both pre- and post-intervention questionnaires. Our baseline findings demonstrated that nurses reported varying levels of confidence in clinical assessment of a DIEP patient. Confidence in managing the donor site was higher, although there was unfamiliarity regarding certain dressings such as Dermabond^TM^ Prineo^TM^. There was a wide range of confidence in the ability to distinguish normal postoperative appearances from potential complications, and many nurses reported uncertainty in answering patients' questions on postoperative garments, showering, and return to driving/ normal activities/ work. Some nursing staff cited a lack of knowledge as a factor in how confident they would feel escalating concerns for review by the surgical team.

**Figure 1:**
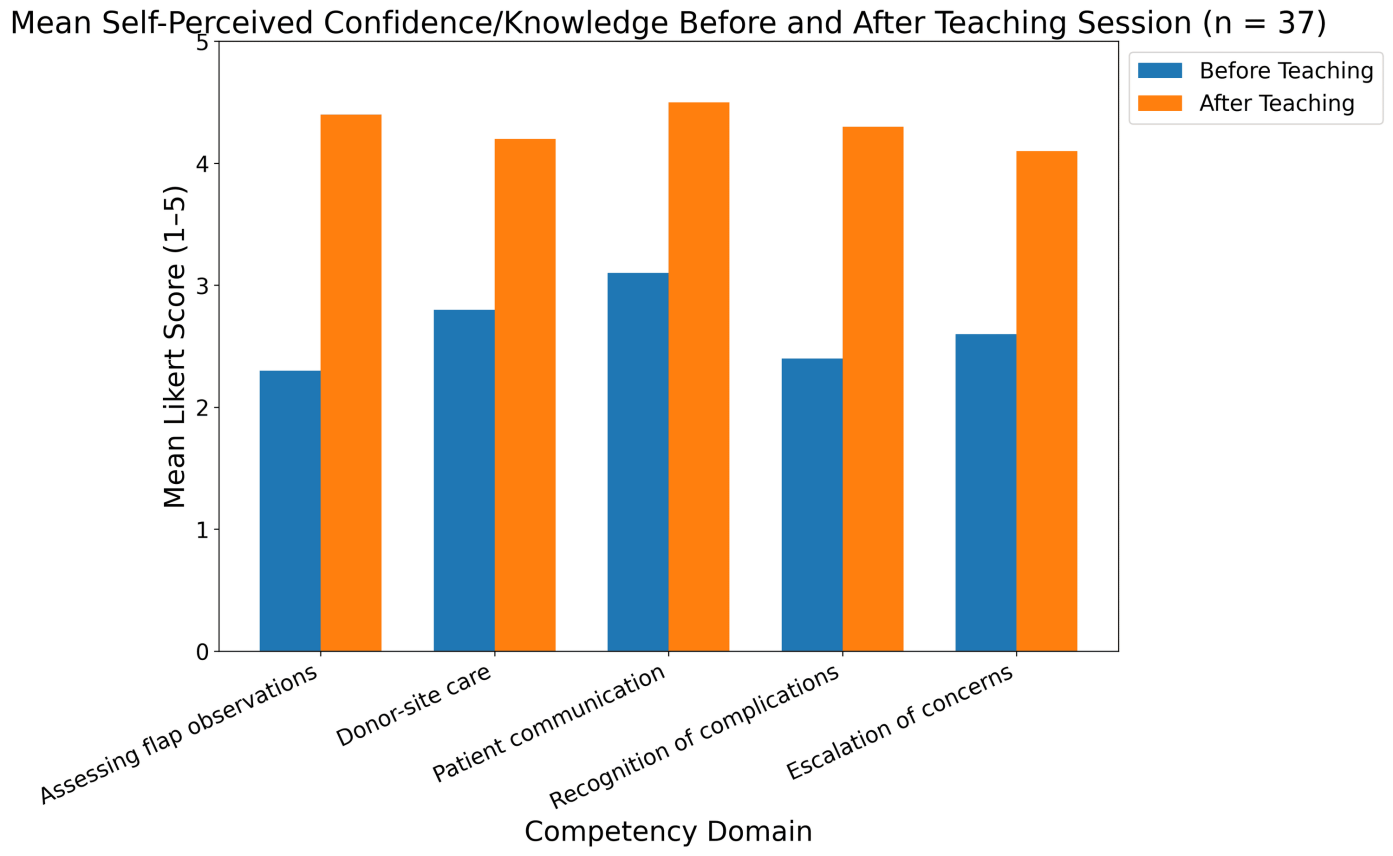
Results

Following the structured educational programme, confidence in clinical assessment of free flaps improved (p<0.001), and all participants reported significant improvement in understanding and confidence across all five domains (Figure 1). There was almost universal (97%) recommendation for the educational programme to be delivered again to other nursing/ outpatient staff.

Participants reported that the teaching programme improved their technical understanding of the vascular anatomy underpinning the deep inferior epigastric perforator (DIEP) flap, particularly the role of perforating vessels arising from the deep inferior epigastric system and the dependence of flap viability on intact microvascular anastomoses. This improved anatomical understanding appeared to translate into greater confidence when assessing postoperative wounds and explaining recovery expectations to patients. From a clinical perspective, this is particularly relevant in the outpatient and dressing clinic setting, where nurses frequently represent the first point of professional contact for patients following discharge. In this context, the ability to confidently interpret normal postoperative findings and provide consistent, evidence-based patient education is essential to supporting safe recovery and reducing unnecessary anxiety or healthcare utilisation.

Nursing staff also reported feeling empowered to adopt a more autonomous role in routine postoperative management when recovery was progressing as expected. At the same time, the teaching programme appeared to provide a structured conceptual framework that enabled participants to recognise and articulate potential deviations from normal healing. This included improved familiarity with the clinical indicators of vascular compromise, wound complications, and abnormal postoperative changes. The development of a shared clinical vocabulary between nursing and surgical teams may represent a particularly important outcome of the intervention. Effective escalation of concerns relies not only on clinical observation but also on the ability to communicate those observations clearly and confidently [2]. In microsurgical reconstruction, where early identification of flap compromise can be critical for successful salvage, strengthening this communication pathway may play an important role in enhancing patient safety.

A further theme identified in qualitative feedback was the perceived improvement in interdisciplinary collaboration. Participants reported that a deeper understanding of the surgical procedure and its postoperative implications allowed them to feel more engaged in the broader reconstructive pathway and patient journey. This aligns with established principles of multidisciplinary care, which emphasise that optimal surgical outcomes depend on coordinated contributions from surgeons, nurses, allied health professionals, and community care teams [2]. By improving nurses' understanding of the reconstructive process, the programme appeared to foster a greater sense of shared responsibility for patient outcomes and reinforce the importance of each professional role within the care continuum.

The opportunity for nursing staff to observe the surgical procedure in the operating theatre was highlighted as one of the most impactful aspects of the educational programme, transforming theoretical concepts into a tangible understanding of surgical anatomy.. Direct observation of microsurgical anastomosis and perforator dissection provided participants with an appreciation of the delicate vascular architecture upon which flap survival depends. For more junior nurses in particular, this experiential exposure helped contextualise the physiological principles underlying postoperative monitoring and wound care. Participants reported that witnessing the technical complexity of the procedure reinforced the importance of factors known to influence flap perfusion, including temperature regulation, avoidance of external pressure on the flap, appropriate patient positioning, and adherence to smoking cessation recommendations [3]. These insights may contribute to a more nuanced understanding of postoperative risk factors and reinforce adherence to established care protocols.

The educational benefits of theatre observation can also be interpreted through the lens of experiential learning theory. Experiential learning emphasises the value of direct engagement with real clinical environments to transform abstract theoretical knowledge into practical understanding [4]. Within surgical education, exposure to operative procedures has been shown to enhance retention of anatomical knowledge and improve comprehension of postoperative management principles [5]. In our programme, theatre observation appeared to serve as a bridge between didactic teaching and clinical practice, enabling participants to visualise the anatomical structures and physiological mechanisms discussed during teaching sessions.

This project demonstrates that structured educational teaching can significantly enhance the clinical competencies and confidence of dressing clinic nurses managing patients following DIEP reconstruction. Although our study did not directly measure clinical outcomes, it is plausible that improved staff knowledge and confidence may facilitate earlier recognition of complications such as venous congestion, arterial insufficiency, fat necrosis, or wound breakdown. Early identification and timely escalation of such complications are critical in microsurgical reconstruction, where delays in intervention may result in increased morbidity or flap loss. We therefore hypothesise that strengthening the educational foundation of outpatient nursing teams may contribute to improved patient safety and postoperative outcomes.

Another important outcome of the intervention was the reported improvement in communication between nursing staff and the surgical team. Effective multidisciplinary communication is a recognised determinant of quality in surgical care and has been associated with improved patient outcomes as well as increased staff satisfaction [2]. Educational initiatives that bring different professional groups into shared learning environments may therefore offer broader organisational benefits beyond the immediate acquisition of clinical knowledge.

Despite these positive findings, several limitations should be considered when interpreting the results of this study. First, the project was conducted within a single centre and involved a relatively small cohort of participants. As a result, the generalisability of the findings to other institutions or healthcare settings may be limited. Second, the primary outcomes measured in this study were based on self-reported confidence and perceived knowledge rather than objective assessments of competence or clinical performance. While perceived confidence is an important determinant of clinical behaviour, it does not necessarily correlate directly with measurable improvements in knowledge or patient outcomes.

Future research should therefore aim to incorporate more objective outcome measures. These might include formal assessments of knowledge acquisition, simulation-based evaluation of clinical decision-making, or analysis of postoperative outcomes such as complication detection rates, readmissions, or flap salvage. Longitudinal follow-up would also be valuable in determining whether the improvements in confidence observed immediately after the intervention are sustained over time. Repeating the educational programme at regular intervals may be necessary to maintain knowledge among existing staff and to support the training of new team members as services expand. Another potential strategy to improve dressing clinic nurses' confidence in a hospital just starting up as a microsurgical unit would be to shadow nurses in other established microsurgery units to share experiences and knowledge.

The introduction of new microsurgical reconstructive services presents unique challenges for healthcare teams, particularly in institutions without prior experience of free tissue transfer procedures, and requires a coordinated educational approach across the wider multidisciplinary team. Our findings highlight the importance of proactively addressing the educational needs of staff responsible for postoperative care. By integrating structured teaching sessions, case-based discussion, and experiential theatre observation, our programme provided a practical and accessible approach to bridging this knowledge gap.

Our quality improvement initiative significantly improved dressing clinic nurses' confidence, knowledge, and ability to deliver optimal postoperative care. We propose that this approach can serve as a practical framework for similar service developments in other plastic surgery units.
